# The Complex of p-Tyr42 RhoA and p-p65/RelA in Response to LPS Regulates the Expression of Phosphoglycerate Kinase 1

**DOI:** 10.3390/antiox12122090

**Published:** 2023-12-08

**Authors:** Oyungerel Dogsom, Amir Hamza, Shohel Mahmud, Jung-Ki Min, Yoon-Beom Lee, Jae-Bong Park

**Affiliations:** 1 Department of Biochemistry, Hallym University College of Medicine, Hallymdaehag-Gil 1, Chuncheon 24252, Kangwon-do, Republic of Korea; oyungerel.d@mnums.edu.mn (O.D.); amirhamza9@hallym.ac.kr (A.H.); slm.btge@hallym.ac.kr (S.M.); jkmin0306@hallym.ac.kr (J.-K.M.); barca9118@hallym.ac.kr (Y.-B.L.); 2 Department of Biology, School of Bio-Medicine, Mongolian National University of Medical Sciences, Ulaanbaatar 14210, Mongolia; 3 National Institute of Biotechnology, Ganakbari, Ashulia, Savar 1349, Dhaka, Bangladesh; 4 Institute of Cell Differentiation and Aging, Hallym University College of Medicine, Hallymdaehag-Gil 1, Chuncheon 24252, Kangwon-do, Republic of Korea

**Keywords:** RhoA phosphorylation, NF-κB, PGK1, superoxide, cancer

## Abstract

Inflammation plays a crucial role in tumorigenesis, primarily mediated by NF-κB. RhoA GTPases are instrumental in regulating the activation of NF-κB. Specifically, the phosphorylation of Tyrosine 42 on RhoA ensures the activation of NF-κB by directly activating the IKKβ associated with IKKγ (NEMO). This study aimed to uncover the molecular mechanism through which p-Tyrosine 42 RhoA, in conjunction with NF-κB, promotes tumorigenesis. Notably, we observed that p-Tyrosine 42 RhoA co-immunoprecipitated with the p-Ser 536 p65/RelA subunit in NF-κB in response to LPS. Moreover, both p-Tyrosine 42 RhoA and p-p65/RelA translocated to the nucleus, where they formed a protein complex associated with the promoter of phosphoglycerate kinase 1 (PGK1) and regulated the expression of PGK1. In addition, p-p65/RelA and p-Tyr42 RhoA co-immunoprecipitated with p300 histone acetyltransferase. Intriguingly, PGK1 exhibited an interaction with β-catenin, PKM1 and PKM2. Of particular interest, si-PGK1 led to a reduction in the levels of β-catenin and phosphorylated pyruvate dehydrogenase A1 (p-PDHA1). We also found that PGK1 phosphorylated β-catenin at the Thr551 and Ser552 residues. These findings discovered that PGK1 may play a role in transcriptional regulation, alongside other transcription factors.

## 1. Introduction

Inflammation is the response of the immune system to detrimental stimuli, such as pathogens, damaged cells and toxic compounds. Acute and/or chronic inflammation responses are attributed to a variety of stimulating factors, which trigger signaling pathways, including the NF-κB, MAPK and JAK-STAT components [1]. Among these pathways, the NF-κB transcription factor plays a pivotal role in inflammation processes [2]. Moreover, NF-kB plays a critical role in regulating various biological processes, including cells’ proliferation, survival, and development, as well as immune responses. The NF-κB family comprises five distinct subunits: p50, p65/RelA, c-Rel, p52 and RelB. Within the nucleus, the p50–p65/RelA heterodimer exhibits greater stability than the other dimers and binds to DNA [3]. However, in the cytosol, the NF-κB dimer associates with the inhibitory protein IκB. The IκB kinase (IKK) complex is composed of the IKKα/IKK1, IKKβ/IKK2 and IKKγ/NEMO subunits. Among these subunits, IKKβ/IKK2 plays the crucial role of phosphorylating IκB, leading to its subsequent degradation. As a result, IKKβ/IKK2 plays a critical role in promoting inflammation in response to proinflammatory stimuli. Of note, the IKKγ/NEMO (nuclear factor-κB-essential modulator) subunit lacks enzymatic activity; instead, it serves as an adapter that bridges the catalytic subunit and the substrate proteins such as IκB [4]. Dysregulation of NF-κB has been linked to various diseases, particularly inflammatory diseases and cancers [5,6]. Notably, reactive oxygen species (ROS) enhance the signaling pathway for the activation of NF-κB [7]. Additionally, Toll-like receptors’ signaling pathways culminate in the activation of NF-κB, which controls the expression of an array of inflammatory cytokine genes [8].

There are numerous proinflammatory agents, such as cytokines, pathogens and ROS. One notable example is lipopolysaccharide (LPS), a constituent of Gram-negative bacteria, which regulates the expression of a wide array of genes through the activation of NF-κB [9]. To delve into the specifics, LPS triggers the activation of Toll-like receptor 4 (TLR4), recruiting MyD88 and subsequently activating TAK1. This activation event leads to the phosphorylation and activation of IKKβ, resulting in the phosphorylation of IκB and its subsequent degradation, ultimately culminating in the activation of NF-κB [8].

In a broad context, Rho GTPase plays a variety of roles in regulating cytoskeletal proteins, cellular morphology, migration and cell proliferation. These activities of Rho GTPases are subject to precise control by specific regulatory factors, including guanine nucleotide exchange factors (GEFs), GTPase activating proteins (GAPs) and the guanine nucleotide dissociation factor (GDI) [10].

The Rho GTPases family, which includes RhoA, Cdc42 and Rac1, has been reported to activate NF-κB [11,12]. In particular, RhoA/Rho-kinase activates NF-kB in the signaling pathway of LPS-induced production of IL-8 in human cervical stroma cells [13]. The molecular mechanism underlying the activation of NF-κB by RhoA involves IKKγ/NEMO, which activates RhoA by facilitating its dissociation from RhoGDI. In response to TGF-β1, RhoA/ROCK, in turn, phosphorylates IKKβ in vitro [14]. Furthermore, oxidized RhoA at the Cys16 and Cys20 residues, along with phosphorylation of the Tyr42 residue, can activate IKKβ by interacting with IKKγ/NEMO. This activation leads to the activation of NF-κB through phosphorylation and degradation of IκB [15,16]. Remarkably, Tyr42-phosphorylated RhoA binds to β-catenin and translocates to the nucleus. In the nucleus, the p-Tyr42 RhoA/β-catenin complex regulates the expression of specific genes, such as vimentin, in response to Wnt3a stimulation [17].

In this study, our objective was to investigate a novel molecular regulatory mechanism involving the regulation of NF-κB’s activation by p-Tyr42 RhoA. Our findings revealed that p-Tyr42 RhoA binds to p-p65/RelA and translocates to the nucleus. Within the nucleus, the p-Tyr42 RhoA/p-p65 complex takes charge of regulating the expression of phosphoglycerate kinase 1 (PGK1).

## 2. Materials and Methods

### 2.1. Materials

Lipopolysaccharide (LPS) was purchased from Sigma-Aldrich (St. Louis, MO, USA). Fetal bovine serum (FBS), Dulbecco’s modified Eagle medium-F12 (DMEM-F12) and penicillin–streptomycin antibiotics were obtained from Cambrex (Verviers, Belgium). The protease/phosphatase inhibitor cocktail was purchased from ApexBio (Boston, MA, USA). Bovine serum albumin (BSA), Nonidet P-40 (NP-40), poly-L-lysine solution (P8920), dichloroacetic acid (DCA), SB-415286 and isopropyl β-D-thiogalactoside (IPTG) were purchased from Sigma-Aldrich Co. (St. Louis, MO, USA). Y27632 was acquired from Millipore-Sigma (Burlington, MA, USA). Skim milk powder (MB-S1667) and LB Broth High Salt (MB-L4488) were obtained from MBcell (SeoCho-Gu, Seoul, Republic of Korea). Alexa Fluor 488 goat anti-mouse IgG, 4′6-diamidino-2-phenylindole (DAPI) and lipofectamine 3000 were obtained from Invitrogen (Carlsbad, CA, USA). ProLong Gold Antifade mounting solution, Alexa Fluor -568 and Alexa Fluor -594 reagents were purchased from Molecular Probes (Eugene, OR, USA). Polyvinylidene difluoride (PVDF) membranes were purchased from Millipore (Billerica, MA, USA). JetPRIME DNA/siRNA transfection reagent was purchased from Polyplus-transfection (Seoul, Republic of Korea). The protein A/G-agarose beads were purchased from Amersham Biosciences (Piscataway, NJ, USA). Anti-β-actin antibodies were purchased from Sigma-Aldrich. Anti-p65, anti phospho-IκB at Ser32/36, anti-RhoA and anti-IKKα/β antibodies were purchased from Abbkine (Wuhan, China). The antibody against p-p65 at Ser536 was purchased from Cell Signaling Technology Inc. (Danvers, MA, USA). The NF-κB p105/p50 polyclonal antibody was purchased from Abbkine (Wuhan, China), and the PGK1 antibody was purchased from Santa Cruz Biotechnology (Santa Cruz, CA, USA). Mouse anti-IKKα/β and anti-IKKγ monoclonal antibodies were purchased from BD Bioscience (Mountain View, CA, USA). Methanol-free formaldehyde was purchased from Pierce (Rockford, IL, USA). Anti-IKKβ antibodies were purchased from Upstate (Lake Placid, NY, USA). RhoA antibodies were purchased from Santa Cruz Biotechnology (Dallas, TX, USA). p-Tyr42 RhoA antibodies were derived in our laboratory using the RhoA peptide epitope of 37-TVFEN (p-Y42) VADIE-47 for immunization. Secondary antibodies of goat anti-rabbit and goat anti-mouse IgG conjugated to HRP were purchased from Enzo Life Sciences (Farmingdale, NY, USA). The sequences of the si-RNAs obtained from Bioneer (Daejeon, Republic of Korea) were as follows: si-RhoA (customized sequence; sense strand, 5′CAGUAUUUAGAAGCCAACU-3′ and antisense strand, 5′-AGUUGGCUUCUAAAUACUG-3), p47 phox (customized sequence; sense strand, 5′-CCGUCCAUGUACCUGCAAA-3′ and antisense strand, 5′-UUUGCAGGUACAUGGACGG-3′) and PGK1 (customized sequence; sense strand, 5′-UCUGGUUAGCUUCGUCACU-3′ and antisense strand, 5′-AGUGACGAAGCUAACCAGA-3′). However, we did not receive information on the si-RNAs purchased from Santa Cruz (Santa Cruz, CA, USA).

### 2.2. Cell Cultures

Human embryonic kidney cell lines (HEK293T), mouse breast cancer cell lines (4T1) and murine macrophage cell lines (RAW264.7) were purchased from the American Type Culture Collection (ATCC). Cell lines were incubated in high-glucose Dulbecco’s modified Eagle’s medium (DMEM) (Biowest USA, Lakewood Ranch, FL, USA) containing 5% fetal bovine serum (Biowest USA, Lakewood Ranch, FL, USA) and 1% penicillin/streptomycin (Lonza, Basel, Switzerland) and grown at 37 °C in a humidified atmosphere of 5% CO_2_. We attempted to determine whether the changes in protein expressions induced by LPS stimulation are a common occurrence across unrelated cell types, such as HEK293, 4T1 and RAW264.7 cells.

### 2.3. Western Blot Analysis

HEK293T, 4T1 and RAW264.7 cells were harvested and then washed twice with ice-cold phosphate-buffered saline (PBS). The cells were then lysed in a RIPA lysis buffer (50 mM Tris-HCl (pH 7.4), 150 mM NaCl, 0.5% NP-40 and 1 mM MgCl_2_), which contained a protease inhibitor cocktail (Sigma-Aldrich, St. Louis, MO, USA), as well as 1 mM NaF and 1 mM Na_3_VO_4_. After lysis, the total protein content was quantified using the BCA protein assay kit (Pierce, Rockford, USA). Approximately 25–30 μg of the total protein was assayed by 8–14% sodium dodecyl sulfate-polyacrylamide gel electrophoresis (SDS-PAGE) and then transferred to a PVDF membrane (Millipore, Billerica MA, USA). The membranes were blocked with 10% (w/v) skim milk for 1 h at room temperature and then incubated overnight at 4 °C with the primary antibodies. Appropriate secondary antibodies (goat anti-rabbit and goat anti-mouse IgG conjugated with HRP) were incubated for 1 to 2 h at room temperature, and then the signals were detected using a chemiluminescence reagent (Millipore, #WBKLS0500, Burlington, MA, USA) with an enhanced chemiluminescence imaging system (Vilber Lourmat Fusion FX, Collegien, France).

### 2.4. Immunoprecipitation

The cells were washed with 1× PBS, and the cell lysates were prepared using a cell lysis buffer (20 mM Tris (pH 7.4), 120 mM NaCl and 1% Nonidet P-40) containing 1 μg/mL each of a protease inhibitor cocktail (Sigma-Aldrich, St. Louis, MO, USA), 1 mM NaF and 1 mM Na_3_VO_4_. The lysates were pre-cleaned with 20 μL of protein A/G beads for 1 h and then used for immunoprecipitation with specific antibodies and a control for normal IgG overnight at 4 °C. Next day, 30 μL of protein A/G beads was added and incubated for 4 h. Subsequently, the beads were collected and washed three times with a washing buffer. Finally, the protein-coated beads were mixed with a 5× sample buffer and boiled for 15 min, and the resulting supernatant was collected for electrophoresis with 8–14% SDS-PAGE.

### 2.5. ROS Assay

When the cells were ready for the experiment, they were stimulated with the selected treatment in serum-free medium. After stimulation, the cells were washed and then fixed in 4% formaldehyde for 15 min at room temperature (RT). To detect ROS, hydroethidine (50 μM in DMSO) was added to the cells and washed two times with 1× PBS. Then the red fluorescence of ethidium was observed under a fluorescence microscope using a filter with an emission wavelength above 590 nm and an excitation filter of 540–552 nm.

### 2.6. Preparation of Cytosolic and Nuclear Fractions

HEK293T cells were stimulated with LPS for various periods and harvested in ice-cold 1× PBS. The harvested cells were then resuspended in s cytoplasmic extract (CE) buffer (20 mM HEPES (pH 7.4), 10 mM KCl, 1 mM MgCl_2_, 0.1% Triton X-100% and 20% glycerol) and incubated on ice for 5 min. After incubation, the mixture was vortexed occasionally and centrifuged at 15,000× *g* for 15 min. The resulting supernatants were collected as the cytosolic fractions. The remaining pellets were subsequently resuspended in an equal volume of nuclear extract (NE) buffer (20 mM HEPES (pH 7.4), 1 mM EDTA, 400 mM NaCl, 0.1% Triton X-100 and 20% glycerol) and incubated on ice for 10 min. The mixture was again vortexed occasionally and centrifuged at 14,000 rpm for 15 min at 4 °C. The resulting supernatant was used as the nuclear extract. Finally, the fractions were mixed with a 5× sample buffer and boiled for 8 min at 95 to 100 °C. After cooling down, the samples were analyzed by immunoblotting.

### 2.7. Small Interference siRNA Transfection

HEK293T cells were seeded at 50% confluency in 5% growth media for 1 day. The cells were then transfected with small interference siRNAs using the HiPerFect transfection reagent. To prepare the transfection mixture, si-RNA (100 ng/mL) was mixed with 4 μL of HiPerFect transfection reagent for 10 s using a vortex. The mixture was incubated for 5–10 min at room temperature, then added dropwise onto the cells in 1 mL of serum-free media and incubated for 4–5 h. Subsequently, the cells were cultured in 2 mL of the appropriate culture medium containing serum and antibiotics at 37 °C and 5% CO_2_ for 48 h. The following small interfering RNAs (si-RNA) were used: si-RhoA (sc-29471, Bioneer #387-3), si-p65 (sc-29411), si-PGK1 (sc-36216, Bioneer #18655-2), si-ROCK2 (sc-36433), si-p47phox (Bioneer #653361-2) and control si-RNA (sc-37007), all purchased from Santa Cruz (Santa Cuz, CA, USA) and Bioneer (Daejeon, Korea). These si-RNAs were transfected at a final concentration of 100 nM.

### 2.8. Site-Directed Mutagenesis

HA-p65 WT, S536A, S536D, HA-RhoA WT, HA-RhoA Y42E and HA-RhoA Y42F mutants were prepared by using a site-directed mutagenesis kit (Intron Biotechnology, Sungnam, Republic of Korea). pCNS RelA proto-oncogenes and NF-κb subunits (Human cDNA clone) were obtained from the Korean Human Gene Bank. Additionally, GST-beta-catenin WT, S502A, T551A and S552A mutants were generated through the use of a site-directed mutagenesis kit (Intron, #15071). 

### 2.9. Chromatin Immunoprecipitation (ChIP) and ChIP-PCR

The cells were stimulated with LPS, then crosslinked with formaldehyde (final concentration: 0.75%) for 15 min at room temperature. Glycine was added to the media with shaking for 5 min at RT to stop crosslinking. The cells were then rinsed twice with 10 mL of cold PBS. The samples were homogenized, and crosslinked chromatin was sheared to <1000 bp fragments by sonication in the ChIP lysis buffer (RIPA buffer). The nuclei were harvested and disrupted by sonication for 20 s, repeated four times. p-Tyr42 RhoA and p-p65 antibodies were incubated overnight with the DNA fragment protein complex and precipitated using protein A/G beads. The beads were washed, and the bound DNAs were eluted using an elution buffer (1% SDS and 100 mM NaHCO_3_). RNA and proteins were removed by incubation with RNAse and proteinase K. Finally, DNA was purified via phenol-chloroform extraction. The DNA fragments were used for sequencing and PCR with primers. DNA sequencing was performed by Ebiogen (Seoul, Republic of Korea), and PCR primers for PGK1 (Pgk1/NM_008828.3: mouse chromosome X, 105, 230, 318-105, 230, 703: forward, 5′-AGGCGCCTGGGAATTCTACCG-3′; reverse, 5′-ACCCACCCCTTCCCAGCCTCTGA-3′) were synthesized by Bioneer (Daejeon, Republic of Korea). The ChIP assay was performed by following the Abcam protocol (Abcam, Cambridge, UK).

### 2.10. Confocal Microscopy

Cells were cultured in 4-well dishes, which were covered by small glasses, and then treated with LPS (10 µg/mL). Subsequently, the cells were fixed with 4% paraformaldehyde for 10 min, neutralized with 20 mM glycine for 10 min, and washed three times with PBS containing 0.1% Triton X-100 (TPBS). The samples were incubated overnight at 4 °C with the specified primary antibodies (1:100), including anti-p-Tyr42 RhoA, -p50, p-Ser536 p65, -β-catenin and -PGK1 antibodies, followed by through rinsing. Subsequently, the antibodies were detected using Alexa Fluor 488-conjugated (green emissions) or Alexa Fluor 568-conjugated secondary antibodies (red emissions) for 2 h at room temperature. DAPI (1 μg/mL) was added for 10 min, and the mounting was stained overnight. The images were observed and recorded using confocal microscopy (LSM 780NLO, Carl Zeiss) with Zeiss Zen 3.7 software.

### 2.11. Cell Migration Assay

4T1 and HEK293T cells were seeded in 6-well plates and allowed to reach >90% confluence. After removing the cell debris by creating a scratch with a sterile 1 mL tip and washing with PBS, the cells were incubated for 48 h in growth media containing the control or the treatments. Photograph documentation of the wounded area was performed at 0 h and 48 h using a digital camera (Nikon D5100, Tokyo, Japan).

### 2.12. Purification of Recombinant Protein

Recombinant GST-β-catenin was expressed in *E. coli* using the host vector pGEX-4T1. Protein expression was induced by adding 0.5 mM isopropylthio-galactosidase (IPTG) to the *E. coli* BL21-transformed culture. The GST-β-catenin fusion protein was purified using glutathione (GSH)-Sepharose 4B beads.

### 2.13. Measurement of Cell Proliferation with MTT Reagents

HEK293 cells were plated in 12-well plates at a density of 1 *×* 10^5^ cells per well or in 96-well plates at a density of 1 *×* 10^3^ cells per well. Prior to the addition of LPS (1 µg/mL), the cells were serum-starved for 6 h. The viability of living cells was assessed using the CCK-8 reagent from the Quanti-Max-WST-8 cell viability assay kit (Biomax, #QM 2500, Seoul, Korea) and MTT reagent (Sigma-Aldrich #M5655, St. Louis, MO, USA), which induced colorization. Subsequently, the optical density (OD) values were measured at 450 nm using a spectrophotometer (Spectramax plus384, San Jose, CA, USA).

### 2.14. Statistical Analysis

The intensity of all protein bands were measured by Photoshop CC2018 (Adobe, San Jose, CA, USA), and GraphPad Prism Version 4.03. GraphPad Software, San Diego, CA, USA) was used for all statistical comparison and analyses. The data are presented as the mean ± standard error of the mean (SEM). All experiments were performed independently at least in triplicate. Data were analyzed by a two-tailed Student’s *t*-test. We assessed statistical significance by considering *p*-values below the specified limits (* *p* < 0.05, statistically significant; ** *p* < 0.01; *** *p* < 0.001, surely significant). The correlations of gene expression were evaluated by Pearson’s product–moment correlation coefficient (ρ).

## 3. Results

### 3.1. LPS Induces the Production of Superoxide through RhoA/ROCK, Leading to the Activation of NF-κB 

**Figure 1:**
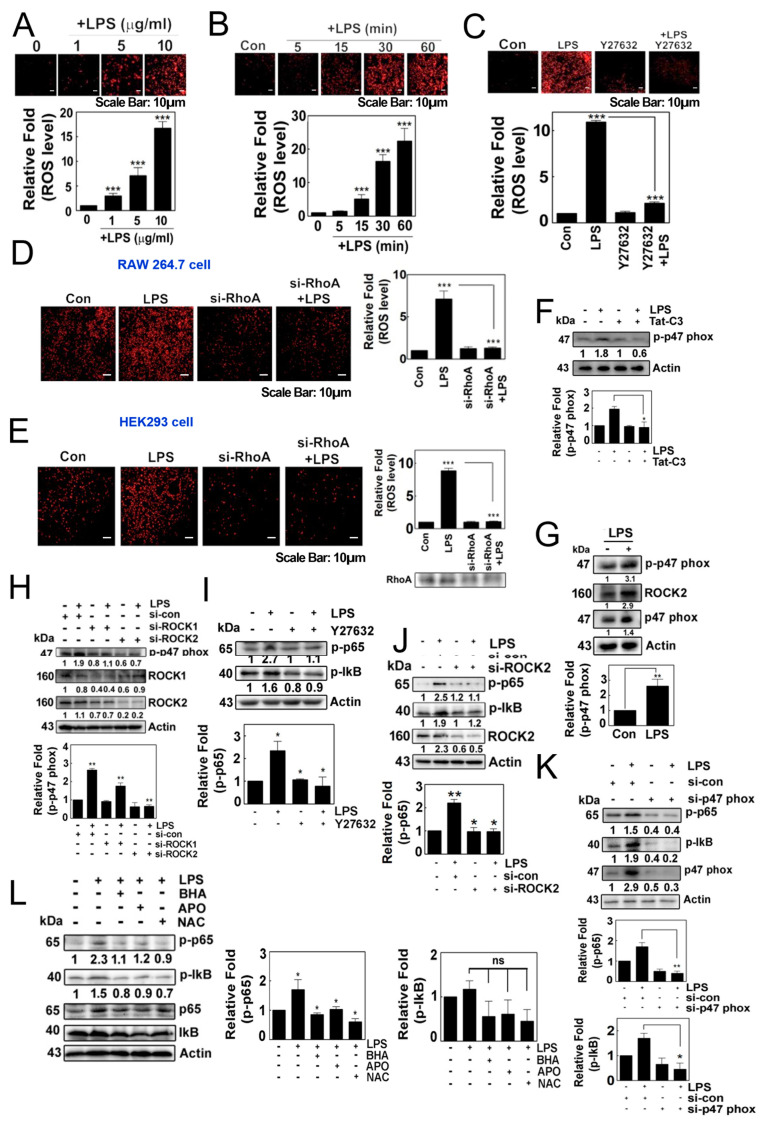
(**A**–**C**) Representative image of the cellular production of ROS/superoxide in HEK293T cells using the dihydroethidium (DHE) assay. ROS/superoxide levels were measured in response to LPS treatment in a concentration-dependent (**A**) and in a time-dependent manner (**B**). Additionally, the production of ROS/superoxide was assessed after pretreatment with Y27632 (10 µM) for 1 h, followed by treatment with LPS (**C**). (**D**,**E**) Superoxide levels were measured after the knockdown of RhoA by si-RNA for 48 h, followed by LPS stimulation in RAW264.7 cells (**D**) and HEK293T cells (**E**). The production of superoxide was visualized using 10 μM hydroethidine (DHE) for 20 min and detected using a fluorescence microscope (Axiovert 200, Carl Zeiss). (**F**) 4T1 cells were cultured without serum for 7 h then pretreated with Tat-C3 (1 μg/mL) for 1 h and stimulated with LPS (10 μg/mL) for 1 h. Protein levels of p-p47phox were measured by Western blotting. (**G**) 4T1 cells were cultured without serum for 8 h and then stimulated with LPS (10 μg/mL) for 1 h. Protein levels of p-p47phox, ROCK2 and p47phox were measured by Western blotting. (**H**,**J**,**K**) Various protein levels were measured by Western blot analysis in HEK293T cells stimulated by LPS after si-RNA mediated knockdown, including ROCK1 and ROCK2 together (**H**), ROCK2 alone (**J**) and p47phox (**K**). (**I**,**L**) Similarly, the levels of p-p65 and p-IκB proteins were measured by introducing inhibitors to the HEK293 cells by 1 h of pretreatment with 10 µM Y27632 (**I**), and a group of ROS scavengers such as 10 µM BHA, 10 µM APO and 10 mM NAC (**L**) in response to LPS. Representative Western blot images are shown and the bands’ intensities were measured by ImageJ software version 10. Data represent the mean ± SD of three independent experiments (* *p* < 0.05; ** *p* < 0.01; *** *p* < 0.001) unless otherwise noted. Western blot data are representative of at least three independent experiments.

LPS induced the production of superoxide in HEK293T cells in a concentration- and time-dependent manner (Figure 1A,B). Of note, Y27632, a ROCK inhibitor, impeded the production of superoxide, suggesting that ROCK may be involved in the response to LPS (Figure 1C). Additionally, si-RhoA abolished the generation of superoxide in both RAW264.7 cells and HEK293 cells in response to LPS (Figure 1D,E). Tat-C3, an inhibitor of Rho GTPases, also prevented the phosphorylation of p47phox (Figure 1F). LPS promoted the phosphorylation of p47phox at the Ser345 residue, a component of NADPH oxidase, accompanied by increases in the levels of p47phox and ROCK2 (Figure 1G). In line with this result, si-ROCK1 and si-ROCK2 attenuated the levels of p-p47phox (Figure 1H). These findings suggest that RhoA/ROCK2 may play a role in regulating the generation of superoxide, likely through p47phox phosphorylation in response to LPS, which aligns with a previous study indicating that the phosphorylation of p47phox and the activation of ROCK by RhoA are critical for the formation of superoxide [18].

Meanwhile, Y27632 attenuated the levels of p-Ser536 p65 and p-Ser32/Ser36 IkB in response to LPS (Figure 1I). Consistent with this result, si-ROCK2 prevented an increase in the levels of p-p65 (Figure 1J). Additionally, si-p47phox significantly repressed the levels of p-p65 and p-IkB protein in response to LPS (Figure 1K). Moreover, the use of butylated hydroxyanisole (BHA), apocynin and N-acetyl cysteine (NAC) to quench the superoxide generated by LPS attenuated the levels of p-p65 and p-IκB protein (Figure 1L). These findings collectively suggest that NADPH oxidase-produced superoxide is required for the activation of NF-κB.

### 3.2. Phosphorylated p65/RelA at the Ser536 Residue Binds with p-Tyr42 RhoA

**Figure 2:**
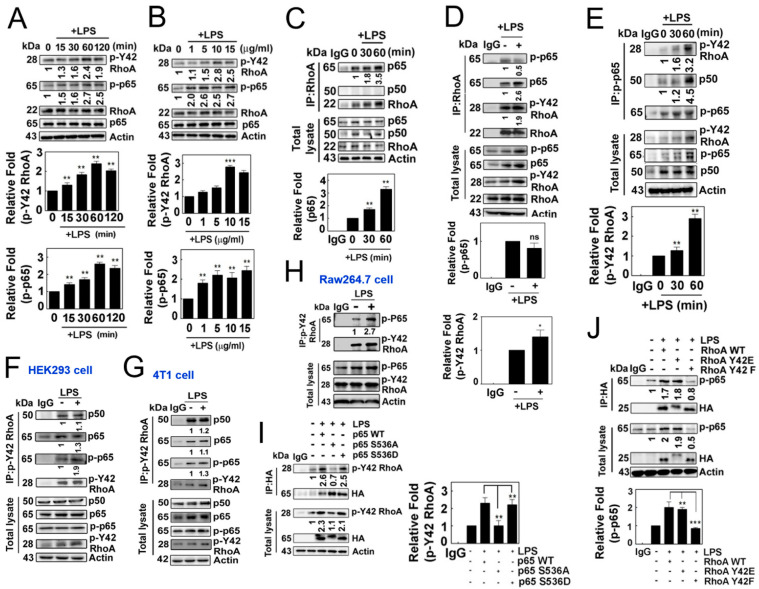
(**A**,**B**) Representative Western blotting images depicting the expression levels of RhoA, p-Y42 RhoA, p65 and p-p65. RAW264.7 cells were stimulated with LPS after serum deprivation, demonstrating both time-dependent (**A**) and concentration-dependent (**B**) responses. (**C**,**D**) LPS stimulated cells were lysed, and the protein lysates were immunoprecipitated using RhoA antibodies, followed by visualization by Western blot analysis. Representative blots are presented for the time course of LPS (**C**) and 1 h of LPS treatment (**D**). (**E**) Similar to Panel C, immunoprecipitation (IP) was performed using p-p65 antibodies, and the resulting proteins were visualized by Western blot analysis. (**F**–**H**) Immunoprecipitation was conducted using p-Y42 RhoA in HEK293T cells (**F**), 4T1 cells (**G**) and RAW264.7 cells (**H**). (**I**,**J**) HEK293T cells were transfected with wild-type p65, as well as mutant S536A and S536D (2 μg/mL each), representing both the phosphomimetic and dephosphomimetic forms (**I**). Similarly, wild-type RhoA, Y42E and Y42F (2 μg/mL each) in both the phosphomimetic and dephosphomimetic forms were transfected (**J**). After 24 h, the cells were cultured in serum-depleted media for 8 h and subsequently stimulated with LPS. Protein lysates were immunoprecipitated using HA antibodies, and the procedure mentioned earlier was followed. The data are the mean ± SD of three independent experiments (* *p* < 0.05; ** *p* < 0.01; *** *p* < 0.001) in case without particular remark. Western blot data are representative of at least three independent experiments.

Next, we observed that LPS induced an increase in p-Tyr42 RhoA as well as p-p65 in a concentration- and time-dependent manner (Figure 2A,B). Notably, we also observed that RhoA co-immunoprecipitated with p65/RelA in response to LPS, but not with p50 (Figure 2C), while LPS did not promote the co-immunoprecipitation of RhoA with p-Ser 536 p65 (Figure 2D). In contrast, p-Ser536 p65 particularly co-immunoprecipitated with p-Tyr42 RhoA along with p50 (Figure 2E). Furthermore, p-Tyr42 RhoA co-immunoprecipitated with p-Ser536 p65, and vice versa, in HEK293, RAW264.7 and 4T1 cells, suggesting that the interaction between p-Tyr42 RhoA and p-p65 may be a prevalent phenomenon in several cell types (Figure 2F–H). Of note, p-Tyr42 RhoA co-immunoprecipitated with p50 even without LPS stimulation (Figure 2F,G). Next, we attempted to clarify whether the phosphorylation of RhoA and p65 is crucial for their protein interactions. The p65 S536A mutant, which mimics the dephosphorylated form, could not co-immunoprecipitate with p-Tyr42 RhoA, while p65 WT and p65 S536D, mimicking the phosphorylated form, readily co-immunoprecipitated with p-Tyr42 RhoA, demonstrating that the p-Ser536 residue of p65 is essential for its interaction with p-Tyr42 RhoA (Figure 2I). Moreover, RhoA Y42F did not co-immunoprecipitate with p-p65, while RhoA WT and RhoA Y42E, mimicking the phosphorylated form, co-immunoprecipitated with p-p65, suggesting that the p-Tyr42 residue of RhoA is critical for its interaction with p-p65 protein (Figure 2J).

### 3.3. p-Ser536 p65/RelA and p-Tyr42 RhoA Translocate to the Nucleus in Response to LPS Stimulation

**Figure 3:**
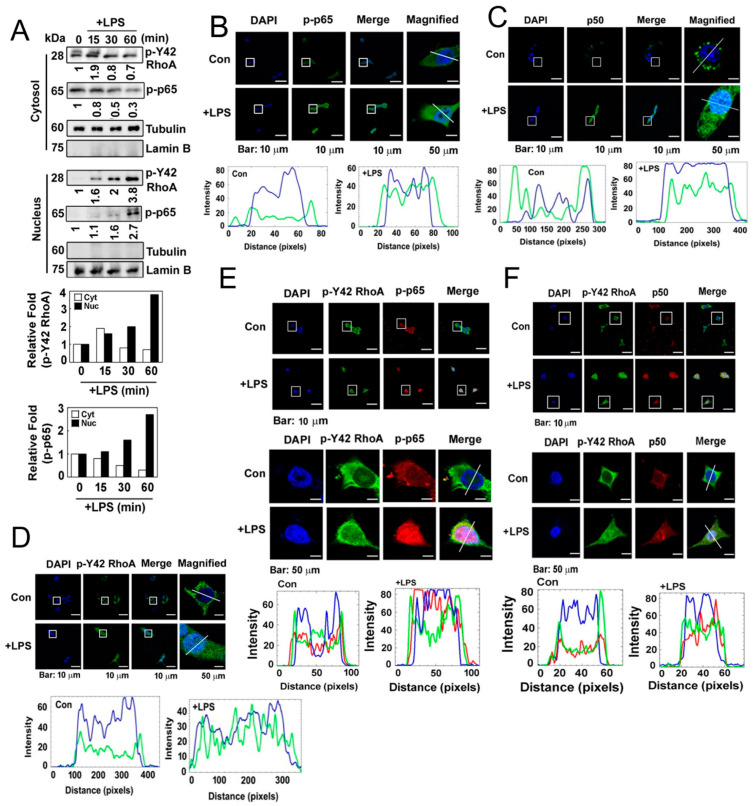
(**A**) HEK293T cells were cultured in a serum-depleted media for 8 h and stimulated with (10 μg/mL) LPS in a time-dependent manner. Nuclear and cytosolic proteins were separated by a fraction assay, and the levels of the target proteins were measured by Western blot analysis. Lamin B and tubulin served as markers for the nuclear and cytosolic fractions. (**B**) HEK293T cells were cultured without serum for 8 h and then treated with LPS (10 µg/mL) for 1 h. Immunofluorescence staining was used to assess the localization of p-p65 (green) and the nucleus (blue). (**C**) Immunofluorescence staining was used to assess the localization of p50 (green) and the nucleus (blue). (**D**) Immunofluorescence staining was used to assess the localization of p-Y42 RhoA (green) and the nucleus (blue). (**E**,**F**) Co-localization was examined by immunostaining with both p-Y42 RhoA (red) and p-p65 or p50 (green), with DAPI used for staining the nucleus (blue). Representative images of the co-localization of p-Y42 RhoA and p-p65 (**E**) and of p-Y42 RhoA and p50 (**F**) are shown. Magnified panels of images of single cells are also shown in both cases. The white lines intersecting the cells serve as indicators for measuring the intensity of fluorescence.

Next, we investigated the localization of the p-Tyr42 RhoA and p-p65 complex in response to LPS. We determined the levels of p-Tyr42 RhoA and p-p65 in the cytosolic and nuclear fractions of LPS-stimulated RAW264.7 cells. We observed that p-Tyr42 RhoA and p-p65 translocated to the nucleus in response to LPS (Figure 3A). Furthermore, when assessed by immunofluorescence in response to LPS, p-p65, p50 and p-Tyr42 RhoA were also found to be localized in the nucleus (Figure 3B–D). Notably, p-Tyr42 RhoA was partially co-localized with both p-p65 and p50 in the nucleus in response to LPS (Figure 3E,F).

### 3.4. LPS Induces PGK1 through p-Tyr42 RhoA and p-65

**Figure 4:**
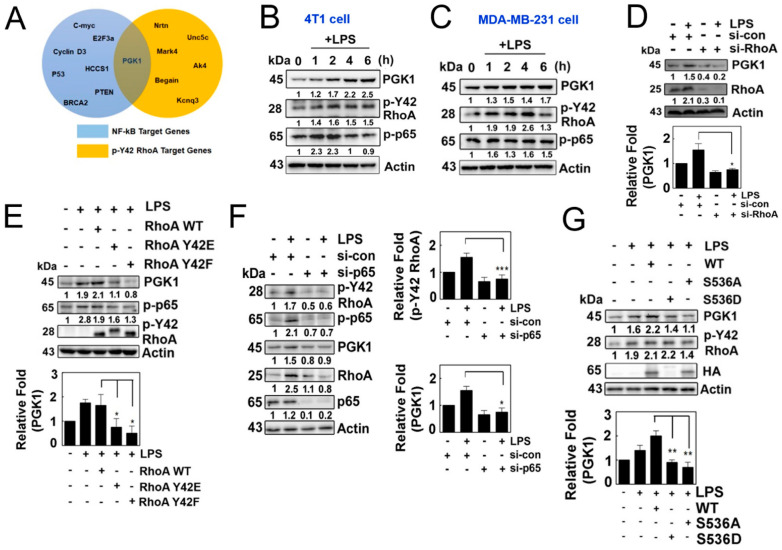
(**A**) Venn diagram depicting the intersection of the target genes of NF-kB and p-Y42RhoA obtained from the ChIP seq database. (**B**,**C**) Cells of interest were cultured in serum-free media for 8 h and subsequently stimulated with LPS in a time-dependent manner. Protein levels of PGK1, p-Y42 RhoA and p-p65 were measured by Western blot analysis, and representative plots for 4T1 cells (**B**) and MDA-MB-231 (**C**) are shown. (**D**) HEK293T cells were transfected with si-RhoA (150 nM siRNA) and incubated for 48 h, and then treated with LPS for 4 h after 8 h of serum starvation. Protein levels were measured by Western blot analysis. (**E**) 4T1 cells were transfected with wild-type RhoA as well as Y42E and Y42F mutants, representing the phosphomimic and de-phosphomimic forms (2 μg/mL each), for 24 h. The cells were then cultured in serum-depleted media for 8 h and stimulated with LPS. Protein levels of PGK1, p-p65 and HA were detected by Western blotting. (**F**) Similar to (**D**), the cells were transfected with p65 siRNA, and the same protocol was used to detect the protein levels. (**G**) Cells were transfected with wild-type p65, as well as mutant S536A and S536D (2 μg/mL each), representing the phosphomimic and de-phosphomimic forms, and then cultured as described in (**E**). Protein levels of PGK1, p-Y42 RhoA, p-p65 and HA were detected by Western blot analysis. Representative Western blot data are presented in each case, and the bands’ intensities were measured using ImageJ software. The data represent the mean ± SD of three independent experiments (* *p* < 0.05; ** *p* < 0.01; *** *p* < 0.001) in case without particular remark. Western blot data are representative of at least three independent experiments.

Based on the aforementioned results, we hypothesized that the p-Tyr42 RhoA/p-p65 complex acts as a transcriptional regulator to control the expression of specific genes. Through ChIP sequencing, we identified specific genes whose promoters were associated with p-Tyr42 RhoA. Furthermore, PGK1 was previously reported as a potential target gene of NF-κB, and the information was obtained from the Boston University website (https://www.bu.edu/nf-kb/gene-resources/target-genes/ accessed on 3 February 2021). Therefore, we selected PGK1 as a common target gene of p-Tyr42 RhoA and NF-κB upon LPS stimulation (Figure 4A). Indeed, we observed that LPS induced the expression of PGK1 in 4T1 and MDA-MB-231 cells, along with an increase in the levels of p-Tyr42 RhoA and p-p65 in a time-dependent manner (Figure 4B,C). Knockdown of RhoA with siRNA attenuated the expression of PGK1 in 4T1 cells (Figure 4D), and the reconstitution of RhoA restored the expression of PGK1, while RhoA Y42F, mimicking the dephosphorylated form, did not rescue its expression (Figure 4E). These findings suggest that RhoA, particularly p-Tyr42 RhoA, is critically required for the expression of PGK1 in response to LPS. Additionally, the knockdown of p65 with siRNA also led to a reduction in the expression of PGK1 and the levels of RhoA protein (Figure 4F). Consequently, the reconstitution of p65 WT restored the expression of PGK1, while p65 S536A, mimicking the dephosphorylated form, did not restore its expression (Figure 4G). These results demonstrated that p-Tyr42 RhoA and p-Ser536 p65 are required for the expression of PGK1 in response to LPS.

### 3.5. p-Ser536 p65/RelA and p-Tyr42 RhoA Bind to the Promoter of the PGK1 Gene

**Figure 5:**
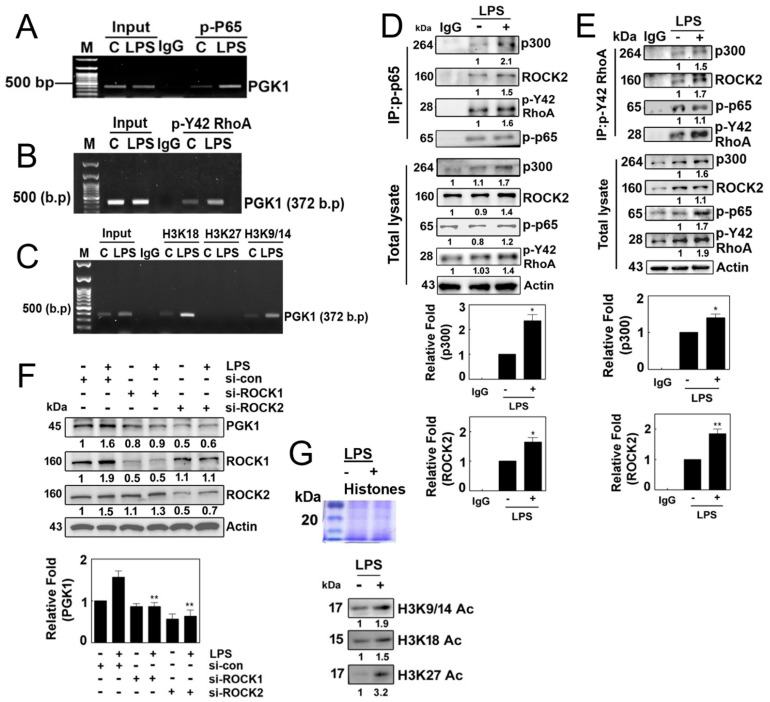
(**A**–**C**) Representative ChIP (chromatin immunoprecipitation) data illustrating the binding of various factors to the promotor region of PGK1 in 4T1 cells. Cells were cultured without serum for 8 h and then stimulated with LPS (10 µg/mL) for 4 h. Immunoprecipitation was performed using specific antibodies, including p-p65 (**A**), p-Tyr42 RhoA (**B**) and acetylated histone (H3K18, H3K27 and H3K9/K14). In each case, normal IgG served as a negative control for the immunoprecipitation assay. (**D**,**E**) Reciprocal immunoprecipitation experiments were conducted with p-p65 (**D**) and p-Y42 RhoA (**E**) antibodies, followed Western blot analysis. HEK293T cells were utilized and cultured in serum-free media for 8 h then stimulated with LPS (10 μg/mL) for 4 h. (**F**) Analysis of 4T1 cells stimulated with LPS (10 μg/mL) for 4 h after si-RNA-mediated knockdown of ROCK1 (100 nM) and ROCK2 (80 nM) for 48 h. Western blot analysis was performed to assess the levels of PGK1, ROCK1 and ROCK2 protein. (**G**) HEK293T cells were exposed to LPS at a concentration of 10 µg/mL, and the levels of acetylated histones (Ac-H3K9/14, Ac-H3K18 and Ac-H3K27) were assessed by Western blotting using extracted histone proteins. The visualization of the histone proteins was achieved via Coomaasie blue staining on SDS-PAGE (upper panel). Representative Western blot data are presented, and the bands’ intensities were quantified using ImageJ software. Data are expressed as the mean ± SD of three independent experiments, with significance denoted as follows: * *p* < 0.05; ** *p* < 0.01.

ChIP-PCR using p-p65 antibodies and primers targeting the PGK1 promoter revealed that p-p65/RelA was associated with the PGK1 promoter (Figure 5A). Furthermore, ChIP-PCR with p-Tyr42 RhoA antibodies and PGK1 promoter-specific primers demonstrated the association of p-Tyr42 RhoA with the PGK1 promoter (Figure 5B). Additionally, ChIP-PCR using Ac-H3K18, Ac-H3K27 and Ac-H3K9/14 antibodies and PGK1 promoter-specific primers indicated the presence of Ac-H3K18 and Ac-H3K9/14 on the PGK1 promoter (Figure 5C). Notably, p-p65 and p-Tyr42 RhoA were found to co-immunoprecipitate with p300 histone acetyltransferase (HAT) and ROCK2 (Figure 5D,E). Knockdown of ROCK1/ROCK2 using siRNAs resulted in reduced expression of PGK1 (Figure 5F). These findings suggest that p-p65 and p-Tyr42 RhoA are associated with the PGK1 promoter, recruiting p300 HAT and ROCK2 to facilitate the acetylation of histone on the PGK1 promoter, ultimately leading to the expression of PGK1. Therefore, we have provided evidence that LPS promotes the levels of histone aceylation, including acetylated H3K9/14, H3K18 and H3K27 (Figure 5G).

### 3.6. PGK1 Leads to the Phosphorylation of β-Catenin during the Process of Inflammation uponLPS

**Figure 6:**
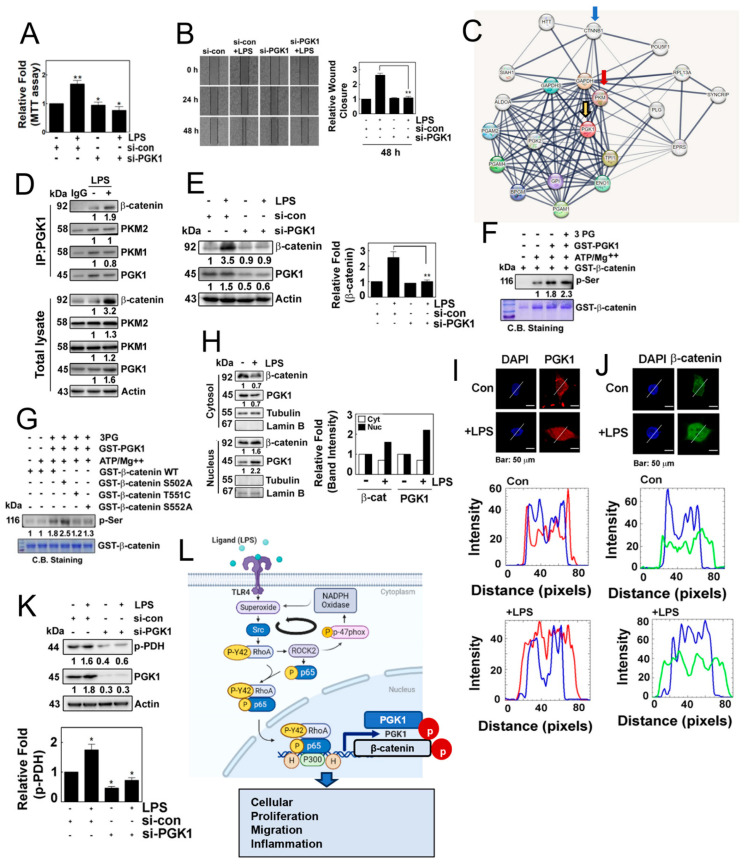
(**A**) Cell proliferation was assessed in HEK293T cells transfected with or without si-PGK1 using the MTT assay. (**B**) Cell migration was evaluated in cells transfected with si-PGK1 for 24 h and 48 h, followed by stimulation with 1 μg/mL of LPS for 24 h, using the wound healing assay. (**C**) Interactome analysis revealed PGK-binding proteins associated with β-catenin. (**D**) Immunoprecipitated PGK1 in HEK293T cells treated with LPS showed increased protein levels in response to LPS. (**E**) HEK293T cells were transfected with si-con or si-PGK1 and treated without or with LPS (10 µg/mL). (**F**) Recombinant GST-PGK1 (1 μg) was incubated with purified GST-β-catenin protein (1 μg) in the presence of ATP (30 μM) and MgCl_2_ (10 mM) and 3-phosphoglycerate (3-PG) (100 μM) for 30 min at RT. Phosphorylated serine (p-Ser) was detected by Western blotting. (**G**) Recombinant GST-β-catenin WT, S502A, T551C and S552A proteins were incubated with the GST-PGK1 proteins released by GSH, in the presence of ATP (30 μM) and MgCl_2_ (10 mM) and 3-PG (100 μM) for 30 min at RT. Then phosphorylated serine (p-Ser) was detected by Western blotting. (**H**) HEK293T cells were treated with or without LPS, and the expression of β-catenin and PGK1 in the cytosol and the nucleus was assessed by western blotting. (**I**,**J**) Co-localization of PGK1 (red) (**I**), and β-catenin (green) (**J**) was determined by immunofluorescence staining, and the nuclei were stained with DAPI (blue). The white lines intersecting the cells serve as indicators for measuring the intensity of fluorescence. Images were captured via confocal microscopy, and plot profiling was performed using ImageJ software. (**K**) HEK293 cells were transfected with 100 nM si-PGK1 (small interfering RNA) and incubated for 48 h. After 8 h of serum starvation, the cells were treated with LPS for 4 h, and the levels of p-PDH protein were assessed via Western blot analysis. (**L**) Schematic illustration of the novel function of the p-Tyr42 RhoA and p-p65 complex in the production of superoxide and inflammation, mediated by transcriptional regulation of PGK1. This illustration was generated using the BioReander.com website. Data are presented as the mean ± SD of three independent experiments (* *p* < 0.05; ** *p* < 0.01) unless otherwise noted. Western blot data are representative of at least three independent experiments.

Next, we aimed to investigate the potential role of PGK1 in the inflammatory process. We observed that si-PGK1 significantly inhibited cells’ proliferation and migration in response to LPS (Figure 6A,B). It is worth noting that PGK1 has been shown to associate with pyruvate kinase M (PKM) [19] and β-catenin [20], according to interactome analyses (STRING: functional protein association network: https://string-db.org, accessed on 2 March 2022) (Figure 6C). Building on this result, we examined the protein–protein interactions in response to LPS. PGK1 co-immunoprecipiated with PKM1, PKM2 and β-catenin, concomitantly increasing co-immunoprecipitation with β-catenin (Figure 6D). Remarkably, the knockdown of PGK1 with siRNA markedly reduced the expression of β-catenin, suggesting that the binding of PGK1 with β-catenin may stabilize β-catenin (Figure 6E). As we hypothesized that PGK1 may phosphorylate β-catenin, leading to its stability, we examined the phosphorylation of β-catenin by PGK1. PGK1 enhanced the levels of phosphorylated β-catenin in the presence of ATP/MgCl_2_ and 3-phosphoglycarate (Figure 6F). At this point, we aimed to identify the specific site of phosphorylation by PGK1on β-catenin. Through an in vitro kinase assay and MALDI-TOF analysis, we identified three candidate phosphorylation sites of β-catenin, including Ser502, Thr551 and Ser552 (Supplemental Figure S1). To confirm which sites of β-catenin were phosphorylated, we generated PGK1 mutants, including S502A, T551C and S552A, and conduced in vitro kinase assay. We found that T551C and S552A mutants attenuated phosphorylation, suggesting that the Thr551 and Ser552 residues of β-catenin may be the phosphorylation sites targeted by PGK1 (Figure 6G). Moreover, LPS induced the translocation of PGK1 and β-catenin to the nucleus, as determined by Western blotting in the separated cytosol and nucleus of HEK293 cells in response to LPS (Figure 6H). Immunohistochemical analyses also revealed the nuclear co-localization of β-catenin and PGK1 (Figure 6I,J). Subsequently, we explored another function of PGK1, and found that si-PGK1 prevented the phosphorylation of PDHA1, suggesting that PGK1 regulates the phosphorylation of PDHA1 in response to LPS (Figure 6K).

## 4. Discussion

In this study, we elucidated the interaction between p-Tyr42 RhoA and the p50/p-Ser536 p65 (RelA) heterodimeric NF-κB complex, and shed light on its regulatory role in nucleus. PGK1 was chosen as a representative gene under the joint regulation of both NF-κB and p-Tyr42 RhoA. Consequently, we aimed to address two key questions: firstly, how p-Tyr42 RhoA regulates the activity of NF-κB within the nucleus, and, secondly, what role PGK1 plays in response to stimulation with LPS. Regarding the first question, our hypothesis posits that p-Tyr42 RhoA plays a role in regulating the acetylation of histone within the NF-κB-binding elements in PGK1’s promoter region. Indeed, p-Tyr42 RhoA recruited p300 histone acetyltransferase (HAT) (Figure 5E), contributing to the acetylation of histone. Additionally, it has been known that p-Tyr42 RhoA activates ROCK2 [17], which, in turn, can phosphorylate p65/RelA (Figure 1J). ROCK2 has been documented to be localized in the nucleus, where it forms an association with p300 HAT, resulting in the phosphorylation of p300 and an increase in its acetyltransferase activity [21]. Moreover, a previous study has shown that p65/RelA interacts with CBP (CREB-binding protein) and p300 HAT [22]. Taking these findings together, we propose that the protein complex consisting of p-Tyr42 RhoA/ROCK2/p-p300/p-p65 stimulates the expression of specific genes. In this study, we specifically focused on PGK1 as a target gene regulated by p-Tyr42 RhoA and p-p65 in response to LPS.

In reference to the various functions of PGK1, recent reports have highlighted its multifaceted roles beyond the conventional metabolic function, which involves the conversion of 1,3-bisphosphoglycerte plus ADP to 3-phosphoglycarate plus ATP. PGK1 has been identified as a protein kinase, a transcription factor coactivator and a disulfide reductase, and it undergoes various posttranslational modifications, including phosphorylation, acetylation, succinylation and ubiquitination [23]. In this study, we proposed that PGK1 may exert an influence on metabolic processes during inflammatory responses. Specifically, PGK1, when phosphorylated at Ser203 by ERK1, translocates to the mitochondria. Here, PGK1 functions as a protein kinase, phosphorylating PDHK1 at Thr338, ultimately resulting in the inactivation of PDH through phosphorylation of Ser293 [24]. Consequently, we explored the impact of the expression of PGK1 in response to LPS on the regulation of the phosphorylation of Ser293 of PHDA1 and demonstrated that si-PGK1 prevented p-PDHA1 (Figure 6K). As a result, the phosphorylation of PDH at Ser293 was observed in response to the expression of PGK1, leading to the inactivation of PDH. This phenomenon resembles the well-known metabolic shift observed in cancer, often referred to as Warburg’s effect. Therefore, we postulate that metabolic changes during inflammation are akin to those observed in cancer, particularly with respect to the activity of PDH.

The expression of PGK1 in colon cancer tissues from metastatic patients increased by 2.6-fold compared with that of patients with no metastasis. Furthermore, PGK1 has been correlated with the increased expression of CYR61, FOS, JUN and EGR1 [25]. Additionally, it has been reported that the heightened expression of PGK1, along with its signaling targets, CXCR4 and β-catenin, in gastric cancer cells promotes peritoneal carcinomatosis [26]. However, the mechanism through which PGK1 regulates the activity of β-catenin has remained largely unexplored. In this study, we discovered that PGK1 not only interacts with β-catenin but also phosphorylates the Thr551 and Ser552 residues within β-catenin (Figure 6G). Very recently, it was reported that *Helicobacter pylori* activates NF-κB, which binds to the CDK1 promoter and induces its expression. Subsequently, CDK1 phosphorylates and inhibits GSK-3β, leading to the activation and accumulation of β-catenin [27]. Although this report revealed that NF-κB induces β-catenin, a different mechanism was observed in this research. As si-PGK1 reduced β-catenin (Figure 6E), we hypothesized that the PGK1 expressed through NF-κB and p-Tyr42 RhoA could stabilize β-catenin through phosphorylation. Recently, it was reported that chitinase 3-like 1 (Chi3l1) binds to CD44 and induces the phosphorylation of β-catenin at Ser552 by Akt in glioblastomas [28] and TANK-binding kinase 1 (TBK1) in cholangiocarcinomas [29], leading to its nuclear translocation. Additionally, PKA phosphorylates β-catenin at Ser675, preventing ubiquitination and consequently promoting the stabilization of β-catenin [30]. Interestingly, Ty654’s phosphorylation of β-catenin facilitates further phosphorylation at Ser675 by PKA [31]. Moreover, fibroblast growth factor receptor 2 (FGFR2), FGFR3, epithelial growth factor receptor (EGFR) and tropomyosin receptor kinase A (TRKA) directly phosphorylate β-catenin at Ty142, leading to an increase in the levels of cytoplasmic β-catenin through the release of β-catenin from the membranous cadherin complex [32]. While it is intriguing that PGK1 may phosphorylate β-catenin at Thr551 and Ser522, the critical function and mechanism of action of this process remain to be fully elucidated. Nonetheless, this discovery provides valuable insights into how PGK1 may influence the regulation of β-catenin.

This study has its limitations in terms of fully understanding how inflammatory LPS enhances tumorigenesis in vivo. However, it does shed light on the molecular mechanism underlying the actions of PGK1 and β-catenin in response to LPS. The inhibition of NF-κB in the upstream and downstream targets by specific inhibitors might be a useful way to treat cancer stem cells [33]. Additionally, PGK1 plays an important role in anti-cancer treatments [34,35,36]. This area warrants further investigations in the future to provide a comprehensive understanding of the mechanisms involved.

## 5. Conclusions

LPS, a well-known proinflammatory agent, was shown to promote the progression of cancer cells by simultaneously activating NF-κB and inducing the phosphorylation of Tyr42 in RhoA, thus facilitating the interaction between these two proteins. Consequently, the resulting protein complex specifically binds to the PGK1 promoter region, leading to the upregulated expression of PGK1. It is worth noting that PGK1 also interacts with β-catenin, influencing its phosphorylation and stability. Although further investigations are required, our findings propose a novel mechanism whereby inflammatory reactions enhance carcinogenesis.

## Data Availability

Data are contained within the article and supplementary materials.

## Supplementary Materials

The following supporting information can be downloaded at: https://www.mdpi.com/article/10.3390/antiox12122090/s1, Figure S1: Identification of candidate amino acids in β-catenin phosphorylated by PGK1.

## Abbreviations

ChIP, chromatin immunoprecipitation; EMT, epithelial mesenchymal transition; GEF, guanine nucleotide exchange factor; GAP, GTPase activating proteins; GDI, guanine nucleotide dissociation inhibitor; GSK-3β, glycogen synthase kinase-3β; HDAC, histone deacetylase; HA, histone acetyltransferase; Luc, luciferase; NAC, N-acetyl cysteine NRX, NF-κB, nuclear factor-κB; nucleoredoxin; NTD, *N*-terminal domain; 3-PG, 3-phosphoglycerate; PGK1, phosphoglycerate kinase 1; PKM, pyruvate kinase M; ROCK, Rho-dependent coiled coil kinase; RhoA Y42E, Tyr42Glu mutant, phospho-mimic; RhoA Y42F, Tyr42Phe, de-phosphomimic form.
