# Supplementary material for: The Complex of p-Tyr42 RhoA and p-p65/RelA in Response to LPS Regulates the Expression of Phosphoglycerate Kinase 1
Source: Antioxidants (Basel). 2023 Dec 8;12(12):2090. doi: 10.3390/antiox12122090 (PMC10740983; doi:10.3390/antiox12122090)
Supplement: Supplementary file 1 [file antioxidants-12-02090-s001.zip › antioxidants-2662760-supplementary.pdf]

Phosphorylation of beta catenin by PGK1

502

490 _ 501	1517.7153	1516.7080	1516.7955	−0.0875	0	K.LLHPPSHWPLIK.A	+ Phospho (ST)
509 _ 521	1389.7013	1388.6940	1388.7346	−0.0407	0	R.NLALCPANHAPLR.E	
529 _ 535	840.5046	839.4973	839.5593	−0.0620	0	R.LVQLLVR.A	

551 552

544 _ 558	1800.8617	1799.8544	1799.6951	0.1593	0	R.TSMGGTQQQFVEGVR.M	+ Oxidation (M); 2 Phospho (ST)
585 _ 605	2428.3479	2427.3406	2427.3526	−0.0119	0	R.GLNTIPLFVQLLYSPIENIQR.V	
619 _ 640	2279.1433	2278.1360	2278.1440	−0.0080	0	K.EAAEAIEAEGATAPLTELLHSR.N	
